# Anti-malarial plants in Ethiopia and their activities on drug-resistant malaria

**DOI:** 10.1093/femsmc/xtac001

**Published:** 2022-01-12

**Authors:** Yimeslal Atnafu Sema, Teshale Areda Waktola

**Affiliations:** School of Biological Sciences and Biotechnolog, Haramaya University, 138 Dire Dawa, Haramaya, Ethiopia; School of Biological Sciences and Biotechnolog, Haramaya University, 138 Dire Dawa, Haramaya, Ethiopia

**Keywords:** anti-malaria, drug resistant, phytochemical constituent, Plasmodium species

## Abstract

In Ethiopia, the impacts of malaria continue to cause a many number of morbidity and mortality that accounts to most-outpatient observations. Ethiopia recently designed to attain nationwide malaria control by 2030 by beginning sub-national elimination in districts with low malaria transmission. However, the rise of drug-resistant parasites, especially *Plasmodium falciparum* hinder the malaria-containment strategies. *Plasmodium falciparum* and *Plasmodium vivax* are dispersed all over Ethiopia, and account for 60% and 40% of malaria cases, respectively. The aim of this report was to overview the phytochemical constituents, diversity, and effect of some compound extracts on drug-resistant plasmodium species. Many plant species, a total 200 identified by 82 studies, are used in traditional malaria treatments throughout the country. *Allium sativum, Croton macrostachyus*, and *Carica papaya* were the more frequently used medicinal plant species. There are so many phytochemical constituents found in medicinal plants used to treat malaria. Alkaloids, flavonoids, phenolics, terpenoids, and glycosides are the most-reported for their effective activity on drug-resistant malaria.

## Introduction

About 60% of the population of Ethiopia is living in the risky areas, mainly areas that lie below 2000 m above sea level. The western part of Oromia, Amhara, Tigray, and Southern Nation and Nationality and Peoples' region (SNNP), and most of the entire areas of Benishangul Gumuz and Gambella regions represent the major malarial hotspots in the country. Up to 2030, Ethiopia has planned to eradicate malaria throughout the country. However, the existence of drug-tolerant Plasmodium species has highly influenced the health and economy of Ethiopia. Specially, *Plasmodium falciparum* has developed resistance to all currently used anti-malarial drugs.

The emergence of malaria species that can resist drugs has led to the findings of many anti-malarial drugs with significant structural difference. Including quinines, triterpenes, sesquiterpenoids, quassinoids, limonoids, alkaloids, lignans, and coumarins are some of the drugs. Medicinal plants are the center of many scientists aimed at discovering optional anti-malarial drugs in many parts of the globe. In Ethiopia, 80% of the population is using different varieties of medicinal plants to treat malaria. The aim of this review is to overview the diversity, phytochemical constituents, and activities of some Ethiopian anti-malarial plant compounds on drug-resistant Plasmodium species.

## Malaria in ethiopia

In Ethiopia, the influence of malaria keeps to cause a substantial number of death that can be considered for most outpatient visits (Esayas *et al*. 2020). Malaria has been one of the major causes of sickness and deaths in Ethiopia (Esayas *et al*. 2020). About 60% of the population is living in the malaria risky, which lie below 2000 m above sea level (Esayas *et al*. 2020). The western lowlands of Oromia, Amhara, Tigray, and SNNP, and almost the entire areas of Benishangul Gumuz and Gambella region are the major malarial hotspots in Ethiopia (Saxena *et al*. 2003).

According to Solomon *et al*. (2020), *P. falciparum* and *Plasmodium vivax* are the widely abundant malaria parasites in Ethiopia, that are accounted for 60% and 40% of malaria cases, respectively. *Plasmodium malariae* accounts for less than 1% whereas *Plasmodium ovale* is rare. The way of transmission for the parasites is a mosquito vector known as *Anopheles arabiensis*. Ethiopia has recently aimed to attain nationwide malaria eradication by 2030 (FMOH 2017).

### Drug-resistant malaria

In Ethiopia, nine studies reported the treatment failures in malaria. The emergence of drug-resistant malaria has raised concerns on the potency and the healing effectiveness of the existing anti-malarial drugs (Abera, Ashebir and Basha 2019). Multidrug-resistant species is present in all malarious areas of Ethiopia (Hailemeskel *et al*. 2019). Chloroquine-resistant *P. vivax* malaria has also been reported from Ethiopia. The emergence of drug-resistant parasites, mainly *P. falciparum* hinder the malaria containment strategies (Koche *et al*. 2016). Among the species, *P. falciparum* has developed resistance to nearly all available anti-malarial drugs. The advent of *P. vivax* resistant to chloroquine and primaquine may, in time, result in a resurgence of vivax malaria as has been seen with *P. falciparum* (Bloland and World Health Organization 2001).

### Ethiopian medicinal plants used to treat malaria

When we see the long history of Ethiopia, up to 80% of the people use traditional medicine, because of the cultural acceptability of healers and local traditional medicines, those medicines do have a relatively low cost and the people cannot easily access modern health facilities (Abera *et al*. 2019). Ethiopian traditional healers is not only concerned with curing of diseases but also with the protection and advertising of human physical, spiritual, social, mental, and material security.

Most of the communities of Ethiopia use many medicinal plants for the healing of malaria, mainly in rural communities of the country. According to Alebie *et al*. (2017), in collective, 82 researches screened a total 200 different plants used in traditional malaria treatments in Ethiopia. Table 1 indicates common medicinal plants applied to heal malaria. *Allium sativum, Croton macrostachyus*, and *Carica papaya* were the most repeatedly used plant species that are registered by ethnobotanical study in Ethiopia for malaria treatment (Abera *et al*. 2019).

The anti-malarial plant species identified in different regions of Ethiopia classified in to 71 different plant families including Fabaceae, Lamiaceae, Euphorbiaceae, Asteraceae, Cucurbitaceae, Solanaceae, Rubiaceae, Aloacea, Acanthaceae, Moraceae, Brassicaceae, Capparidaceae, Asclepiadaceae, Anacardiaceae, Apocynaceae, Apiaceae, Malvaceae, Meliaceae, Rutaceae, Ranunculaceae, Rosaceae, Menispermaceae, and Verbnaceae (Table 1).

**Table 1.** Medicinal plants used to treat malaria in Ethiopia.

Number	Scientific name of plants	Family name	Local name	Part(s) used	Method of preparation
1	*Allium sativum*	Alliaceae	Nech shinkurt(A)	Bulb	The bulb taken with ‘injera’ and *Capsicum annuum* L. for 5 days before eating breakfast.
2	*Carica papaya*	Caricaceae	Paappaayyaa	Leaf/root	When the leaves become yellow, it will be dried, powdered, and boiled in water and then a cup of boiled leaf powder will be taken for 5 days.
3	*Lepidium sativum*	Brassicaceae	Shinfaa(O),feetoo(A)	Seed	Dried seed, powdered and eaten with injera to get cure from malaria or rubbed on the body for protection from mosquito bite.
4	*Vernonia amygdalina*	Asteraceae	Eebicha(O)	Leaf	Leaves crushed and soaked in water and the exudates drunk orally for 5 days.
5	*Justicia schimperiana*	Acanthaceae	Sensel(A)	Leaf	Crush and squeeze, then drink with with coffee.
6	*Aloe weloensis*	Aloaceae	Eret tafa(A)	Latex	Crush and mash, then drink with Tella.
7	*Croton macrostachyus*	Euphorbiaceae	Mekanisa(O)	Fruit, leaf, and bark	Crush and powderize, then drink with water.
8	*Lobelia gibberoa*	Lobeliaceae	Jibara(A)	Root	Crush and powderize, then drink with water.
9	*Phytolacca dodecandra*	Phytolaccaceae	Mehan Endod (A)	Root	Grind, then drink with water.
10					
11	*Cicer arietinum L*.	Fabaceae	Shinbira(A)	Seed	Germinate, then eat the concoction.
12	*Capsicum annuum*	Solanaceae	Karia(A)	Fruit	Chop the concoction, then eat.
13	*Lycopersicon esculentum*		Timatim	Leaf	Squeeze, then drink.
14	*Droguetia iners*	Urticaceae	Yewoba medihanit (A)	Leaf	Chopped and mixed with *Premna oligotricha* and boiled together, one glassful drenched.
15	*Lantana trifolia*	Verbenaceae	Yewoba medihanit	Root	Chopped and soaked with water and mixed with local alcoholic drink (Areke).
16	*Premna oligotricha*	Lamiaceae	Yewoba medihanit (A)	Leaf	Ground and mixed with water.
17	*Zornia glochidiata*	Fabaceae	medihanit (A)	Root and bark	Chopped and boiled/concoction with local drinks and boiled coffee leaf.
18	*Euphorbia abyssinica*	Euphorbiaceae	Kulkual	Root	Crush and drink with milk.
19	*Skebergia capensis sparrm*.	Meliaceae	Lol(A)	Bark	Infusion of fresh pulverized bark.
20	*Urtica simensis*	Urticaceae	Sama(A)	Root	Crushed and dried, then mixed with fresh water, drink one glass of it and drink much amount of milk.
21	*Acacia robusta*	Fabaceae	Wangey(O)	Root	Concoction
22	*Azadirachta indica*	Meliaceae	Kinina(O) neem(A)	Leaf	Extract Leaf

Abera *et al*. (2019).

**Table 2.** Some common phytochemicals found in medicinal plants.

No.	Class of phytochemical	Occurrence as natural product (%)	Role in health care
1	Phenolics	45	Anti-oxidants, anti-cancerous, cytotoxicants, anti-microbials, and vasodilating.
2	Terpenoids and Steroids	27	Anti-microbials, detoxifying agents, strengthners, anti-rheumatics, anti-malarial, and hepaticidal.
3	Alkaloids	18	Neuropharmaceuticals, anti-cancerous, sedatives, anti-microbials, and insecticidal.
4	Other chemicals	10	Anti-inflammatory and immunostimulating.

Koche *et al*. (2016).

**Table 3.** Phytochemical constituents of medicinal plants used to treat malaria in Ethiopia.

Number	*Plant scientific name*	Plant vernacular name	Compound name
1	*Carica papaya*	Paappaayyaa (Afan Oromo)	Alkaloid, flavonoid, tannin, saponin, and phenol
2	*Vernonia amygdalina*	Ebicha (Afan Oromo)	Alkaloid, flavonoid, tannin, saponin, and phenol
3	*Allium sativum*	Nech shinkurt (Amharic)	Alkaloid, flavonoid, tannin, saponin, and phenol
			Terpenoid, anthraquinones, glycosides, and steroids
4	*Justicia schimperiana*	Sensel (Amharic)	Polyphenol, tanins, steroids, anthraquinones, phlobatanins, carotenoids, and xanthoproteins
5	*Croton macrostachyus*	Mekanisa (Afan Oromo)	Polyphenol, tanins, steroids, anthraquinones, phlobatanins, carotenoids and xanthoproteins
6	*Phytolacca dodecandra*	Mehan	Alkaloid, flavonoid, and phenol
		Endod (Amharic)	Terpenoid
7	*Lepidium sativum*	Shinfaa (Afan Oromo) and fetoo (Amharic)	Polyphenol, flavonoids tannins, and saponins
8	*Capsicum annuum*	Karia (Amharic)	Phenol, flavonoid, and proanthocyanidins
9	*Aloe pulcherrima*	Hargisa (Afan Oromo)	Nataloin, 7-hydroxyaloin, three anthraquinones (chrysophanol, aloesaponarin I, and aloesaponarin II)
10	*Aloe debrana Chrstian*	Eret (Amharic)	Aloin
11	*Aloe Sinana*		Microdontin, aloin, and aloinoside
12	*Asparagus africanus*	Seriti (Afan Oromo) and Kestencha (Amharic)	Polyphenols, saponins, phenolic glycosides, phytosteroids, terpens, and alkaloids
13	*Balanites rotundifolia*		Alkaloids and cardiac glycosides
14	*Bersama abyssnica fresen*	Azamir	Tannins, flavonoids, phenolic glycosides, phytosteriods, and saponins
15	*Calpurnia aurea*	Chekata (Afan Oromo) and digita (Amharic)	Alkaloids, cardiac glycosides, flavonoids, phenols, phytosteriods, saponins, terpenoids, and tannins.
16	*Croton macrostachyus*	Bissana (Amharic) and bekenisa (Afan Oromo)	Alkaloids, saponins, phenolic compounds, cardiac glycosides, terpenoids, and flavonoids
17	*Dodonaea angustifolia*	Ketketa	Pinocembrin, the flavanol santin, and the clerodane diterpene
18	*Echinops kebericho*	Kebercho	Sesquiterpenoids, monoterpenoids, α-Guaiene and β-santalene and monocyclic sesquiterpenoid hydrocarbon β-elemene
19	*Gnidia Stenophylla Gilg*	Kataricha (Afan Oromo) and demer Erit (Amharic)	Flavonoids, chromones, lignans, neolignans, and phenolic compounds
20	*Jatropha curcas*	Ayderke	Alkaloids, phenolic groups, flavonids, saponins, steroids, tannins, cardiac glycosides, and terpenoids
21	*Strychnos mitis*	Yedingamst (Amaric), mulqaa, and satto (Afan Oromo)	Alkaloids, tannins saponins, flavonoids, terpenoids, steroid, phenols, and glycosides
22	*Otostegia integrefolia*	Tinjut	Flavonoids, phenols, terpenoids, saponins, steroids, and glycosides
23	*Withania somnifera*	hanzo (Afan Oromo) and gizawa (Amharic)	Tannins, alkaloids, polyphenols, flavonoids, phytosterols, phenolic glycosides, polyphenol, sphytosteroids, and saponins

Abera *et al*. (2019); Muhammed and Idris (2019); Nduche *et al*. (2019); Irene *et al*. (2012); and Rizwan *et al*. (2015).

As shown in Table 3, many remedies are prepared from the leaf and parts of the medicinal plants. The reason why many traditional practitioners use the leaf parts, compared to others for remedial preparation is due to their accessibility and to prevent them from extinction (Okello and Kang 2019). Because, collecting the root parts of those plants for preparation of traditional medicines has negative impacts on the future life of the plants.

According to Wadood *et al*. (2013), anti-malarial herbal medicines were mainly given through oral route (82.7%), while rarely given via nasal (5.5%), and whole body (2.8%). And also fresh solid remedies were also eaten and chewed directly upon collection. For the meantime, powdery materials were smoked and applied *via* nasal (Wadood *et al*. 2013).

### Common phytochemicals found in traditional medicinal plants

Typically, the plant chemicals have been categorized as primary or secondary metabolites, based on their function to the plant. The primary metabolites includes the common sugars, amino acids, proteins, purines and pyrimidines of nucleic acids, chlorophylls, and so on, whereas the secondary metabolites are the leftover plant chemicals including alkaloids, terpenes, flavonoids, lignans, plant steroids, curcumines, saponins, phenolics, and glucosides (Alebie *et al*. 2017).

Plant chemicals are chemical substances that are manufactured by plants, to help them resist pathogens and pests. When the plants are consumed without knowing of their cellular actions or mechanisms, plant chemicals can be poisoned the peoples (Ntie-Kang *et al*. 2014).

According to Wadood *et al*. (2013), the primary compounds include Chlorophyll, proteins, and common sugars and the secondary compounds include terpenoids, alkaloids, and phenolic compounds. The class of terpenoids are useful in attracting beneficial mites. Alkaloids are applied as anaesthetic agents. Alkaloids are applied as anaesthetic agents that are present in medicinal plants. Terpenoids are useful to prevent inflammation, cancer, malaria, over cholesterol synthesis, and virus and bacterial activities. Terpenoids are also crucial in attracting useful mites and consume the herbivorous insects.

### Phytochemical constituents of Ethiopian anti-malarial traditional medicinal plants

According to Abera *et al*. (2019), each plant may have different chemicals. Those different chemicals of medicinal plants used to treat malaria. The table finalizes most of the plant chemicals that are obtained from the Ethiopian anti-malarial medicinal plants.

The plants bioactive compounds, particularly the bitter principles (alkaloids and terpenoids), indicate their application in the making of traditional medicines against malaria, fever, and inflammation (Koche *et al*. 2016). Plants are known to be a rich reservoir of bioactive secondary metabolites, and are both obtained from plant origin (Muiva-Mutisya *et al*. 2014).

### Activities of phytochemicals on some drug-resistant malaria

The occurrence of drug-resistant Plasmodium species has affected the health and economic status of the population. This has imitated the scientists to discover many anti-malarial remedies with significant structural varieties, including quinines, triterpenes, sesquiterpenoids, quassinoids, limnoids, alkaloids, lignans, and coumarins. Plants have been identified as a healthy source for drug discovery and interestingly, the secondary compounds that are obtained from plants and are still clinically used now a days in the treatment of severe malaria (Pinheiro *et al*. 2019).

Anti-malarial activities of the most common compounds on some drug-resistant parasites are briefly described below.

- Alkaloids: several are positive against CQ-sensitive and CQ-resistant strains of *P. falciparum in vitro* (Moges and Moges 2019). Alkaloids are involved in resistance reversal (Rasoanaivo *et al*. 2011).
- Flavonoids: are previously seen as a promising class of NPs exhibiting anti-malarial and anti-plasmodial activities (Moges and Moges 2019).
- Phenolics: found to be active against chloroquine-sensitive (D6) and chloroquine-resistant (W2) strains of *P. falciparum* (Ramanandraibe *et al*. 2008).
- Terpenoids: are inhibitant of the drug-resistant *P. falciparum* (Goulart *et al*. 2004).
- Glycosides: the glycosides only showed weak to moderate anti-plasmodial activities against the D6- and W2-resistant strains of *P. falciparum* (Moges and Moges 2019).

## Conclusion

Around 80% the population of Ethiopia uses traditional medicines because the people accept traditional healers and local pharmacopeias, the low cost of traditional medicine as we compared it the modern one, and also there is a limitation to get modern health services. Traditional healers in different parts of the country used many species of plants for the treatment of malaria. In this report, 24 anti-malarial medicinal plant species are listed. This report showed that, there is a potential for isolation of compounds effective against drug-resistant malaria from medicinal plant extracts. These plants consist of different types of phytochemicals that can be used to produce effective anti-malarial drugs. These chemicals naturally exist in the medicinal parts that have a defensive role against various diseases. A large number of compounds, which have anti-malarial property found in 15 plants are reported in this report. Alkaloids, flavonoids, phenolics, terpenoids, and glycosides have potential effect on some drug-resistant Plasmodium strains.

## Acknowledgements

This review would not have been possible without the support of many people. Many thanks to my friends Kelemu Mulluye and Firya Girma who read my numerous revisions and helped make some sense of the confusion. Also thanks to my colleague, Sultan Seyida and Zinab Sherafu, who offered guidance and support.

## Supplementary Material

xtac001_Supplemental_Files
