# Supplementary material for: Anti-malarial plants in Ethiopia and their activities on drug-resistant malaria
Source: FEMS Microbes. 2022 Jan 12;3:xtac001. doi: 10.1093/femsmc/xtac001 (PMC10117797; doi:10.1093/femsmc/xtac001)
Supplement: xtac001_Supplemental_Files [file Author_Response_to_Decision_Letter.pdf › Author_Response_to_Decision_Letter.pdf]

Authors' Response to Decision Letter

Comments and responses
Response of reviewer 1 comments
Minor point comment
Comment 1 : page2, Line 12, instead of P. falciparum, better write Plasmodium falciparum
Correction 1 : Corrected as "Plasmodium falciparum" page 1, line 6.
Comment 2 : Page 3. Line 12-13: "Ethiopia has currently designed to achieve nationwide malaria eradication by the coming 2030" Modified the line, the statement needs to be reframed.
Correction 2: Modified into "Up to 2030 Ethiopia has planned to eradicate malaria throughout the country". page 1, line 23
Comment 3: Page 3, Line 17/ Page 3, line 23, there is a spacing problem.
Correction 3: Adjusted.
Comment 4 : Page 3, line 21-23, needs modification.
Correction 4: Rewritten as " The emergence of malaria species that can resist drugs has led to the findings of many anti-malarial drugs with a significant structural difference. Including quinines, triterpenes, sesquiterpenoids, quassinoids, limonoids, alkaloids, lignans, and coumarins are some of the drugs" .page 1, line 28.
Comment 5 : Page 3, line 45, what is 'regionin'??.
Correction 5: Corrected as " region is" page 2 line 12
Comment 6 : Page 3, line 54, the word 'transmition' is spelled incorrectly
Correction 6: Changed into " transmission "
Comment 7: Page 4, line 10, avoid repetition of words i.e., 'existing'
Correction 7: One of it was avoided on page 2 line 21
Comment 8: Page 4, line 19, please check to space.
Correction 8: Checked and corrected
Comment 9: Page 4, line 30-32: Not written adequately
Correction:9 Corrected .
Comment 10 : Page 5, line 10, 'existing antimalaria' sounds inappropriate, please reframe the complete statement.
Correction10 : It was reframed
Comment 11 : Page 8, line 25, "materials were given mouth directly" sounds incorrect.
Correction 11 : Deleted
Comment 12 : Page 8, line 21-27, the statement are not correctly written
Correction 12 : Corrected .
Major points to be considered.
Comment 1 : I strongly encourage authors to provide structures for hit compounds (Phyto-constituents from Allium sativum, Croton macrostachyus and Carica papaya etc……), may be a separate Figure
Correction 1 : I couldn't find structures for hit compounds.
Comment 2 : The subtitle on page 9, "Common phytochemicals found in traditional medicinal plants" may not be given importance as it provides very general information, may be reduced to a couple of sentences. Instead, chemical structure for suitable phytochemicals, and their MOA, etc. could be valued
Correction 2 : The number of sentences is not too much (it is three sentences).
Comment 3 : In the absence of sufficient information on "treating drug-resistant malaria by plant-based materials", the title of the article "resistance malaria" sounds inadequate. Authors are advised to expand this portion and include rationale how these phytochemicals/extracts are able to treat resistant malaria with proper citations
Correction 3 : You can get the information under the title "Activities of phytochemicals on some drug-resistant malaria".
Response of reviewer 2 comments
Comment 1 : Table 3: Would it be possible to add a column on the potency of phytochemical compounds as anti-malarial? This would be useful information on whether these plant chemicals are useful starting points for drug development
Correction 1 : The compound that was listed in table 3 is phytochemicals that do have anti-malarial activities.
Comment 2 : In the section for "Activities of phytochemicals on some drug-resistant malaria", the authors provide five broad classes of phytochemicals that work against the different types of drug-resistant malaria parasites. Would it be possible to have a summary table of the potency of these

compounds against malaria parasites?
Correction 2 : It was mentioned under table 3.
Comment 3 : “Among the species, P. falciparum has developed resistance to nearly all available antimalarial drugs (CDC, 2018).” Could the CDC reference please be replaced by some more relevant reviews that cover this aspect?
Correction 3 : Corrected
Comment 4 : The relatively low cost of traditional medicine and difficult access to modern health facilities (Wikipedia, 2019). – Could the authors please replace the Wikipedia reference with more relevant papers that support this statement?
Correction 4 : Replaced
