# Supplementary material for: Anti-malarial plants in Ethiopia and their activities on drug-resistant malaria
Source: FEMS Microbes. 2022 Jan 12;3:xtac001. doi: 10.1093/femsmc/xtac001 (PMC10117797; doi:10.1093/femsmc/xtac001)
Supplement: xtac001_Supplemental_Files [file FEMSMC-2021-037_Decision_Letter.pdf › FEMSMC-2021-037_Decision_Letter.pdf]

29-Sep-2021
Manuscript ID: FEMSMC-2021-037
Title: Anti-malarial plants in Ethiopia and their activities on drug resistant malaria
Authors: Sema, Yimeslal; Waktola, Teshale
Submitted to: FEMS Microbes

Dear Mr. Sema,

Thank you for submitting the above manuscript to our journal. It has now been expertly refereed and recommendations are given below.

You will see that the reviewers, although they found merit in your manuscript, have raised a number of concerns that preclude acceptance in its present form. If you feel able to address these comments below, then I will be happy to consider a revised manuscript.

Please return your revised manuscript by 28-Nov-2021 (EDT).

Always upload your revised manuscript via Manuscript Central. You can create your revised manuscript from here, by clicking on this link *** PLEASE NOTE: This is a two-step process. After clicking on the link, you will be directed to a webpage to confirm. ***

https://mc.manuscriptcentral.com/femsmc?URL_MASK=3f179904b8f14abda0f45800d31afa5c.
Alternatively you can log in to your submitting author centre and use the link "click here to submit a revision".

Please remember to adhere to the author guidelines when preparing the revision of your manuscript (https://academic.oup.com/femsmicrobes/pages/General_Instructions). Importantly, this includes a detailed, point-by-point response to the comments of the reviewers and incorporating a track changed version of your revised manuscript.

FEMS Microbes aims for a fair and transparent peer review system. We encourage authors to publish reviewer reports with the author's responses alongside their final published article as Supplementary Material. Please note that this optional. The reviewer of your manuscript will have the option to include their name on the published paper. For more information see
https://academic.oup.com/femsmicrobes/pages/General_Instructions#Transparent%20Peer%20Review.

We recommend Peerwith, a platform for author services, in case you require support with the preparation of your manuscript. Peerwith eases the process of revising your manuscript by connecting academics with relevant experts who offer a suite of author services including English language editing, figure enhancement and manuscript formatting. You can use the following link to connect with experts via Peerwith (https://www.peerwith.com/fems/request/femsmicrobes).

Once the submission process is complete, you will receive an email confirmation and the manuscript will be passed automatically to your Editor.

If your paper is accepted, the electronic files will in principle be used for final publication, but the publisher may request a set of high-quality print-outs of your figures for production purposes.

I am looking forward to receiving your revision in due course. Please let me know if you decide not to submit a revision, or cannot respond within the given time.

Best wishes,

Dr. Kimberly Kline
Editor-in-Chief
FEMS Microbes

Sign up for our TOC alerts http://ow.ly/EEsC1

Associate Editor's Comments to Authors:

Reviewers' Comments to Authors:

Reviewer: 1

Comments to the Author
Despite manuscript provides interesting information referee cannot recommend publication of the manuscript in its current form. Following points need to addressed.
Minor points.
Page 2, Line 12, instead of P. falciparum, better write Plasmodium falciparum.
Page 3, Line 12-13: “Ethiopia has currently designed to achieve nationwide malaria eradication by the coming 2030” Modified the line, the statement needs to be reframed.
Page 3, Line 17/ Page 3, line 23, there is a spacing problem.
Page 3, line 21-23, needs modification.
Page 3, line 45, what is ‘regionin’??.
Page 3, line 54, the word ‘transmition’ is spelled incorrectly.
Page 4, line 10, avoid repetition of words i.e., ‘existing’
Page 4, line 19, please check spacing.
Page 4, line 30-32: Not written adequately.
Page 5, line 10, ‘existing antimalaria’s sounds inappropriate, please reframe the complete statement.
Page 8, line 25, “materials were given mouth directly” sounds incorrect.
Page 8, line 21-27, the statement are not correctly written
Major points to be considered.
I strongly encourage authors to provide structures for hit compounds (phyto-constituents from Allium sativum, Croton macrostachyus and Carica papaya etc……), may be a separate Figure.
The subtitle on page 9, “Common phytochemicals found in traditional medicinal plants” may not be given importance as it provides very general information, may be reduced to couple of sentences. Instead, chemical structure for suitable phytochemicals, and their MOA etc. could be valued.
In the absence of sufficient information on “treating drug resistant malaria by plant-based materials”, the title of the article “resistance malaria” sounds inadequate. Authors are advised to expand this portion and include rationale how these phytochemicals/extracts are able to treat resistant malaria with proper citations.
Writing part is a bit complex to understand. Even simple sentences are written improperly rendering confusion while reading, understanding the manuscript. I look forward a revised manuscript, significantly rewritten.
References are not provided uniformly, must be provided for all following the journal’s guidelines.
Lastly, referee is little nervous to note the following recent papers on plant based antimalarials from Ethiopia, hence authors are advised to revise the manuscript considering originality-hypothesis in mind to avoid duplication of the information.
https://malariajournal.biomedcentral.com/articles/10.1186/s12936-020-03231-7
https://www.researchgate.net/publication/321527504_Antimalarial_activity_of_selected_Ethiopian_medicinal_plants_in_mice
https://link.springer.com/article/10.1186/s12936-017-1953-2
https://www.sciencedirect.com/science/article/pii/S0378874117322997?casa_token=J0r0p8aa5wMAAAA A:kXeZvDd9WQtwa07XpKUazPT8TZu-h0POKrWo_YuyliKtHMySuOFI_avucUpQJlRv-uyS9UGcQzO8 (not cited)

Reviewer 1: Dr. Wai-Hong Tham

Comments to the Author
The review titled “Anti-malarial plants in Ethiopia and their activities on drug resistant malaria” provides a description on plant species used in traditional medicine as malaria treatments. It covers their traditional usage and preparation, classes of phytochemical compounds and anti-malarial activities on drug resistant malaria parasites.

Table 3: Would it be possible to add a column on the potency of phyto-chemical compounds as anti-malarials? This would be useful information on whether these plant chemicals are useful starting points for drug development.

In the section for “Activities of phytochemicals on some drug resistant malaria”, the authors provide five broad classes of phytochemical that work against the different types of drug resistance malaria parasites. Would it be possible to have a summary table of the potency of these compounds against malaria parasites?

Referencing:
1. “Among the species, P. falciparum has developed resistance to nearly all available anti-malarial drugs (CDC, 2018).” Could the CDC reference please be replaced by some more relevant reviews that cover this aspect?

2. the relatively low cost of traditional medicine and difficult access to modern health facilities (Wikipedia, 2019). – Could the authors please replace the Wikipedia reference to more relevant papers that support this statement?

In general the review does need substantial editing assistance for grammar, spelling and general formatting. There are too many changes to list in this review.

Date Sent: 29-Sep-2021
